# Targeted Magnetic Nanoparticles for Mechanical Lysis of Tumor Cells by Low-Amplitude Alternating Magnetic Field

**DOI:** 10.3390/ma9110943

**Published:** 2016-11-22

**Authors:** Adi Vegerhof, Eran A. Barnoy, Menachem Motiei, Dror Malka, Yossef Danan, Zeev Zalevsky, Rachela Popovtzer

**Affiliations:** 1 Faculty of Engineering & The Institute of Nanotechnology and Advanced Materials, Bar-Ilan University, Ramat-Gan 5290002, Israel; adivegerhof@gmail.com (A.V.); eabnoy@gmail.com (E.A.B.); motiei.biu@gmail.com (M.M.); yosidanan@gmail.com (Y.D.); zeev.zalevsky@biu.ac.il (Z.Z.); 2 Faculty of Engineering Holon Institute of Technology, Holon 5810201, Israel; drorutah@gmail.com

**Keywords:** biomedical, magnetic field, MRI, cetuximab, head and neck cancer

## Abstract

Currently available cancer therapies can cause damage to healthy tissue. We developed a unique method for specific mechanical lysis of cancer cells using superparamagnetic iron oxide nanoparticle rotation under a weak alternating magnetic field. Iron oxide core nanoparticles were coated with cetuximab, an anti-epidermal growth factor receptor antibody, for specific tumor targeting. Nude mice bearing a head and neck tumor were treated with cetuximab-coated magnetic nanoparticles (MNPs) and then received a 30 min treatment with a weak external alternating magnetic field (4 Hz) applied on alternating days (total of seven treatments, over 14 days). This treatment, compared to a pure antibody, exhibited a superior cell death effect over time. Furthermore, necrosis in the tumor site was detected by magnetic resonance (MR) images. Thermal camera images of head and neck squamous cell carcinoma cultures demonstrated that cell death occurred purely by a mechanical mechanism.

## 1. Introduction

One of the major challenges for cancer therapy is focused destruction of tumor cells without damaging the surrounding environment. Currently available treatments, such as radiotherapy and chemotherapy, can harm healthy tissue as well, while regional hyperthermia generally cannot use high enough temperatures to completely destroy cancer cells, necessitating combination with other therapies. 

Recently, alternating magnetic fields (AMFs) have been suggested for the delivery of thermoablative cancer therapy via activation of targeted magnetic nanoparticles (MNPs) [1]. However, AMFs cause dielectric heating and require high-amplitude and long-duration pulses, which cause nonspecific heating in tissues [1,2]. Theoretically, in a non-homogeneous AMF gradient, MNPs oscillate mechanically and generate ultrasound waves, generating intracellular ultrasounds, which in turn can account for magnetic hyperthermia without any global temperature increase [3].

The present study offers a novel system using low-amplitude AMFs that lyse tumor cells not by heating but by the mechanical force of rotating magnetic nanoparticles (MNPs). MNPs are highly useful for various biomedical applications, as their size can be easily controlled, and they are biocompatible and non-toxic. Superparamagnetic iron oxide MNPs also serve as effective MRI contrast agents. Moreover, as super-paramagnetic materials, their magnetization depends upon an external magnetic field, which prevents their aggregation [4,5]. It has been shown that rotational nanoparticle movement can be used for cell death by injuring the lysosomal membrane structures [6]. It was also hypothesized that the shear forces created by the generation of oscillatory torques of MNPs bound to the lysosomal membranes would lead to membrane permeabilization, causing extravasation of lysosomal content and inducing apoptosis [6]. 

One of the primary challenges for AMF cancer therapy is the development of targeted magnetic particles that can also avoid detection and phagocytosis by the reticuloendothelial system (RES) [5,7], and thereby prolong blood circulation time [7,8]. The rapid RES uptake of nanoparticles can be decreased by using a surface coating of poly(ethylene glycol) (PEG). Nevertheless, the greater part of PEGylated particles reach the liver and spleen after circulation, resulting in lower amounts that reach the tumor. In addition, PEG coating reduces the interaction of nanoparticles with the tumor cells. We attempted to resolve these challenges by coating the MNPs with the tumor-targeting element cetuximab, a chimeric mouse–human monoclonal antibody that binds with high affinity to the anti-epidermal growth factor receptor (EGFR), which is expressed at high levels by various epithelial tumors [9,10]. Cetuximab demonstrates prolonged blood circulation time [9] and promotes EGFR internalization and degradation, yet because its effect is relatively slow [11], it is clinically used in combination with chemotherapy for treating colorectal cancer [12] or with radiotherapy for head and neck cancer [13]. 

In the present work, we examined a new system for cancer therapy based on targeted magnetic nanoparticles that selectively reach the tumor due to cetuximab coating, and are then induced to rotate by external, low-amplitude AMFs, thereby causing mechanical lysis of the cells. Thus, iron oxide MNPs sized 50, 100, and 200 nm were coated with cetuximab and assessed for their efficacy after an application of 4 Hz AMFs in a head and neck squamous cell carcinoma (HNSCC) cell culture and a mouse model for head and neck cancer.

## 2. Methods

### 2.1. MNPs

Magnetite core MNPs (Chemicell GmbH ©, Berlin, Germany) were coated with PEG, consisting of a mixture of thiol-polyethylene-glycol (mPEG-SH) (~85%, MW ~ 5 kDa) and a hetero functional thiol- PEG-acid (SH-PEG-COOH) (~15%, MW ~ 5 kDa). The PEG mixture was added in excess to each solution with different MNP sizes—50, 100, and 200 nm—and then stirred for 4 h at room temperature. The solutions were then centrifuged in order to reach higher concentrations and to remove excess PEG molecules. We activated the molecules in the PEG-MNP solution by adding 1-ethyl-3-(3-dimethylaminopropyl) carbodimide HCl (EDC) (ThermoFisher Scientific, Waltham, MA, USA) and N-hydroxysulfosuccinimide sodium salt (NHS) (Chem-Impex International, Wood Dale, IL, USA), and by stirring the mixture overnight. To specifically target the EGF receptor, we added C225 (Cetuximab, Merck KGaA, Darm-stadt, Germany), conjugated by a covalent bonding of an amine (NH_2_) group to the carboxylic group of SH–PEG–COOH. We used MNPs sized 50, 100, and 200 nm due to the simplicity of their synthesis and based on our previous study [14]. The classification of materials with magnetic properties is based on their magnetic susceptibility (χ), which is defined by the ratio of the induced magnetization (M) to the applied magnetic field (H). The M–H curve shows a hysteresis loop, which is the irreversibility of the magnetization process that is related to the existence of a magnetic domain within the material. The susceptibilities of these materials depend on their atomic structures, their temperature, and the external field H. These materials, such as MNPs, become a single magnetic domain at the nano scale and therefore maintain one large magnetic moment. They display superparamagnetism by the lack of remnant magnetization after the removal of external magnetic fields, which enables the particles to maintain their colloidal stability and avoid aggregation. Thus, these superparamagnetic materials manifest a sigmoidal M–H curve, with no hysteresis. This property makes their use in biomedical applications feasible [15,16,17].

### 2.2. In Vitro Study

Human head and neck squamous cell carcinoma (HNSCC) A431 cells were seeded in 60 mm dishes (1 × 10^6^ cells per dish), with 5 mL of Dulbecco’s modified Eagle’s medium (DMEM) containing 5% fetal calf serum, 0.5% penicillin, and 0.5% glutamine [9]. Nanoparticles of different sizes (50, 100, and 200 nm) were incubated with A431 cells (3 plates for each size) at a final total concentration of 60 μg/mL. After 2 h incubation, the medium was removed and cells were washed three times with PBS to remove excess MNPs. All the in vitro experiments were performed in a biological safety cabinet and maintained at a temperature of 37 °C. Then, the cells were exposed to an alternating magnetic field. AMFs were produced by an electrical system that includes an electro-magnet made of copper coil surrounding an EC Ferrite core, with 30 Ω resistance, connected to a waveform generator that sets the voltage and frequencies provided to the coil. This system was used in our previous study [14]. The electrical parameters used were a 4 V, 4 Hz unipolar magnetic field. Cell viability was measured by a trypan blue assay: 50 μL of cell suspension was taken and mixed with an equal volume of 0.4% trypan blue (Sigma-Aldrich, Rehovot, Israel). The solution was mixed thoroughly and allowed to stand for 5 min at room temperature. Cell viability was determined by counting the unstained (live) cells under a microscope (Leica, Modiin, Israel) [18]. The total number of live cells in a sample at each time point was calculated by counting cells under the microscope in four 1 × 1 mm squares of one chamber and determining the average number of cells per square.

### 2.3. Temperature Elevation

The temperature of the cells was elevated using an electronic hot plate with a monitored temperature. The temperature over the sample was imaged using a radiometric thermal imaging camera with dimensions of 320 × 240 pixels, a temperature sensitivity of 0.07 °C, and a spatial resolution of 0.5 mm (model A325, FLIR Systems Inc., Boston, MA, USA). The camera is sensitive to thermal radiation at a wavelength range of 8 μm–14 μm. Each cell culture dish was recorded for several seconds in ambient temperature (at the center of the laser beam) [19].

To measure the solution temperature in cell solutions with 2 mg/mL–10 mg/mL MNPs and AMFs, we used T-type thermo-couples (Omega Engineering, Inc., Stamford, CT, USA) applied inside the sample vial and secured with polyimide tape.

### 2.4. In Vivo Study

A431 cells (2 × 10^6^) were injected subcutaneously into the back flank area of nude male mice (total *n* = 19) aged 6 weeks. When the tumors reached a diameter of 4 mm–5 mm, mice were divided into four groups. The mice were anesthetized with 10 mg/mL ketamine and 0.2 mg/mL xylazine, and treatment groups (*n* = 5 per group) received an intravenous injection of either 50, 100, or 200 nm MNPs (30 mg mL^−1^; 200 μL, 300 mg per kg body weight; into the tail vein). Control mice (*n* = 4) received an injection of non-conjugated cetuximab (200 μL into the tail vein) without MNPs. All groups were subsequently exposed to the magnetic field (~2 h after injection).

The study was conducted in compliance with the protocols approved by the Institutional Animal Care and Use Committees (IACUC) of Bar Ilan University, Ramat Gan, Israel. The mice were housed in a barrier-controlled facility under the strict care of the veterinarian in charge of the IACUC. Throughout the experiment, mice were continuously monitored for any signs of clinical disease or weight loss. 

### 2.5. Magnetic Field Application

**Figure 1:**
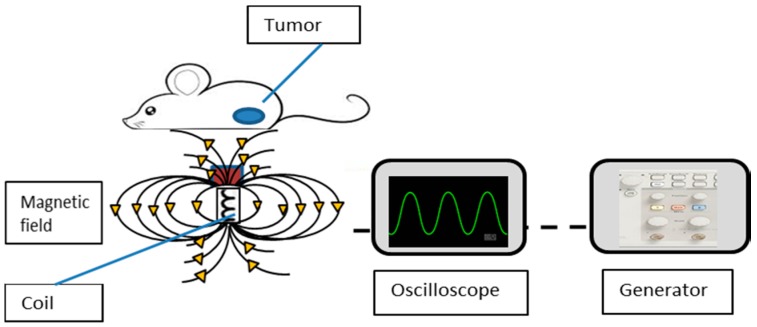
Schematic design of the experimental setup.

To assess the effect of the magnetic field on MNP movement, we performed a preliminary study to determine the optimal electrical parameters for cell destruction and imaging using the speckle method [14]. The electrical setup used in the present work is the same as previously described [14]. The system includes an electro-magnet that is made of a copper coil surrounding an EC Ferrite core. The coil is connected to a waveform generator that sets the voltage and frequencies provided to the coil. The generator is connected to a scope that shows the amplitude and frequency on a screen. The mice are set on the coil, positioning the tumor directly on top of the coil, as schemed in Figure 1. The four groups of mice were directly exposed to 4 V, 4 Hz unipolar AMFs (as schemed in Figure 1) every other day for 30 min, for a total of 7 treatments over 14 days. Smaller exposure levels have been tolerated well in prior experiments [20,21,22]. All mice were housed in a clean room that did not contain any active magnet source. Previous studies have described the mechanical effects of the combination of static and gradient magnetic fields on nanoparticles [23,24]. Briefly, AMFs were carried out by applying 4 V to a constructed coil at a fixed frequency of 4 Hz, with a magnetic field of 6.2 G (measured by Bell 5170 gaussmeter (Berg engineering, Rolling Meadows, IL, USA). The mechanical response of the MNPs to weak AMFs has been shown in our previous work [9]. 

### 2.6. MRI

Magnetic nanoparticles were used as the MRI contrast agents. MNPs are more efficient than the commonly used gadolinium (Gd) chelates, which mostly non-specifically and rapidly accumulate in the liver, thus allowing for only a short time imaging window [12]. In vivo 3D gradient-echo and T2-weighted MR imaging was performed 6 hours after injection of 50, 100, and 200 nm coated MNPs on three mice, using a 1.5 Tesla GE MRI system and the standard phased-array GE head-coil. Gradient-echo MRIs were acquired with a 512 × 512 matrix, 16 × 12 cm^2^ field of view, a repetition time of 425 ms, an echo time of 15 ms, a flip angle of 15°, and 2 mm slices with no gap. T2-weighted fast spin echo MRIs were acquired with a 512 × 512 matrix, 16 × 12 cm^2^ field of view, a repetition time of 5500 ms, an echo time of 80.2 ms, and 2 mm slices with no gap [25]. MNP-conjugated cells generate a strong MRI contrast, which facilitates the image application [23].

### 2.7. Tumor Growth Progression

During the period of the AMF treatments, mice were monitored for tumor growth every other day. Height, width, and depth of the tumor were assessed using calipers, and these parameters were multiplied to calculate the tumor volume. The percentage of tumor growth was calculated by the division of the tumor volume of each mouse after each AMF treatment by the initial tumor size of each mouse, and by averaging the results for each group. All mice were euthanized at the conclusion of the study (Week 3).

### 2.8. Statistical Analysis

We compared the average values of the tumor volume growth percentage of different particle sizes and the control groups by using an analysis of variance (ANOVA). Statistical significance was defined as *p* < 0.05. Additionally, an independent-samples t-test was conducted to compare tumor volumes growth percentage for the 200 nm MNPs and the Cetuximab control group. The sample size values were tested via the ANOVA given in [26]: E = Total number of animals − Total number of groups. Here, the total number of animals was 19, and the total number of groups was 4, yielding an E value of 15, indicating that our sample sizes is suitable for statistical analysis [26].

## 3. Results

### 3.1. Effect of Coated MNPs and AMF Treatment In Vitro

**Figure 2:**
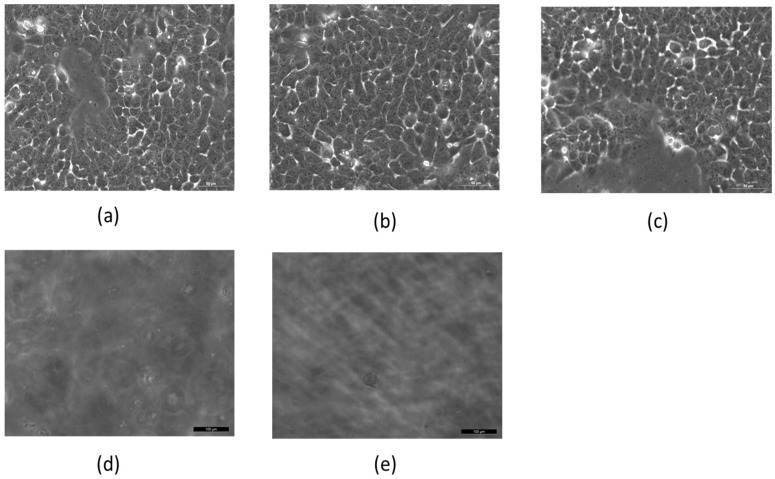
Cell viability of 10^6^ A431 cell cultures after various treatments. (**a**) Untreated cells; (**b**) cells without magnetic nanoparticles (MNPs) treated with 15 min of alternating magnetic fields (AMFs); (**c**) cells incubated with MNPs without AMFs; (**d**) cells incubated with MNPs and treated with 15 min of AMFs; (**e**) cells with MNPs heated for 5 min on a hot plate. Imaged by Leica microscope X20.

First, we tested the effect of MNPs and AMFs on A431 cell viability in vitro. Cells were incubated for 2 h with cetuximab-coated MNPs (50, 100, or 200 nm), and subsequently treated with AMFs (15 min) (*n* = 3 per group). Additional groups (*n* = 3 per group) included untreated cells, cells incubated with the three sizes of coated MNPs without subsequent AMFs, cells not incubated with MNPs but treated with AMFs (15 min), and cells incubated with the three sizes of coated MNPs and subsequently heated above the critical temperature by a hot plate (45 °C, 5 min; without AMF treatment) (Figure 2a–e).

Figure 2 shows results for cells incubated with 50 nm MNPs. Cells with MNPs but not treated with AMFs, and cells without MNPs but treated with AMFs, showed a viability similar to that of untreated control cells (Figure 2a–c). However, cells treated with the combination of MNP incubation followed by AMF treatment resulted in complete cell death (Figure 2d), similar to heated cells (Figure 2e).

Viability tests conducted on cells incubated with 100 and 200 nm MNPs showed results similar to those of 50 nm MNPs. Previous work conducted in our lab demonstrated a gradual decrease in the number of live cells over time, until they reached ~50% of the initial amount by the end of the 5 min AMF treatment [14]. 

**Figure 3:**
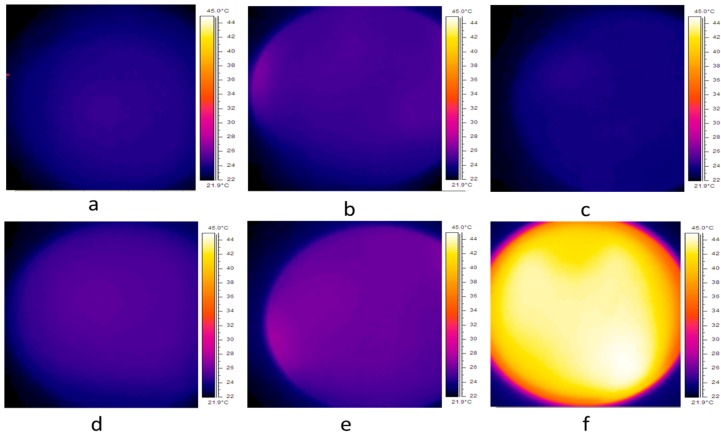
Thermal profile for (**a**) 10^6^ A431 cells only; (**b**) solution of coated MNPs immediately after 15 min of AMF treatment; (**c**) 10^6^ A431 cells after 15 min of AMF treatment; (**d**) 10^6^ A431 cells incubated with coated MNPs after 15 min of AMF treatment; (**e**) 10^6^ A431 cells with coated MNPs after 40 min of AMF treatment and (**f**) 10^6^ A431 cells after 5 min on a hot plate set to 45 °C. Images taken by thermal imaging camera.

To ensure that cell death was caused by AMF-induced particle motion and was not due to hyperthermia, the cells incubated with the various cetuximab-coated MNP sizes were imaged with a thermal camera immediately after AMF treatment. Figure 3 presents the thermal profile for cells incubated with 50 nm MNPs. Untreated control cells showed an average temperature of 23.7 °C (Figure 3a). A solution of coated MNPs treated with AMFs (15 min), and cell cultures (with no MNPs) treated with AMFs (15 min), showed average temperatures of 24.2 °C and 23.5 °C, respectively (Figure 3b,c). Cells incubated with coated MNPs followed by 15 or 40 min of AMF treatment showed an average temperature of 25.3 °C and 26.2 °C, respectively (Figure 3d,e). Cells incubated with coated MNPs and subsequently heated by a hot plate (45 °C for a time period of 5 min) showed an average temperature of 41 °C (Figure 3f). Temperature profiling tests conducted for 100 and 200 nm MNPs showed results similar to those of 50 nm MNPs. Thus, our findings indicate that cell death was not caused by high temperatures but rather by the mechanical motion of MNPs induced by AMFs. This was supported by Hapuarachchige et al. [23], who showed that the ferromagnetic resonance frequency is typically in the range of ~10^8^–10^10^ Hz, and this mechanism does not contribute to the heating produced by AMFs.

**Figure 4:**
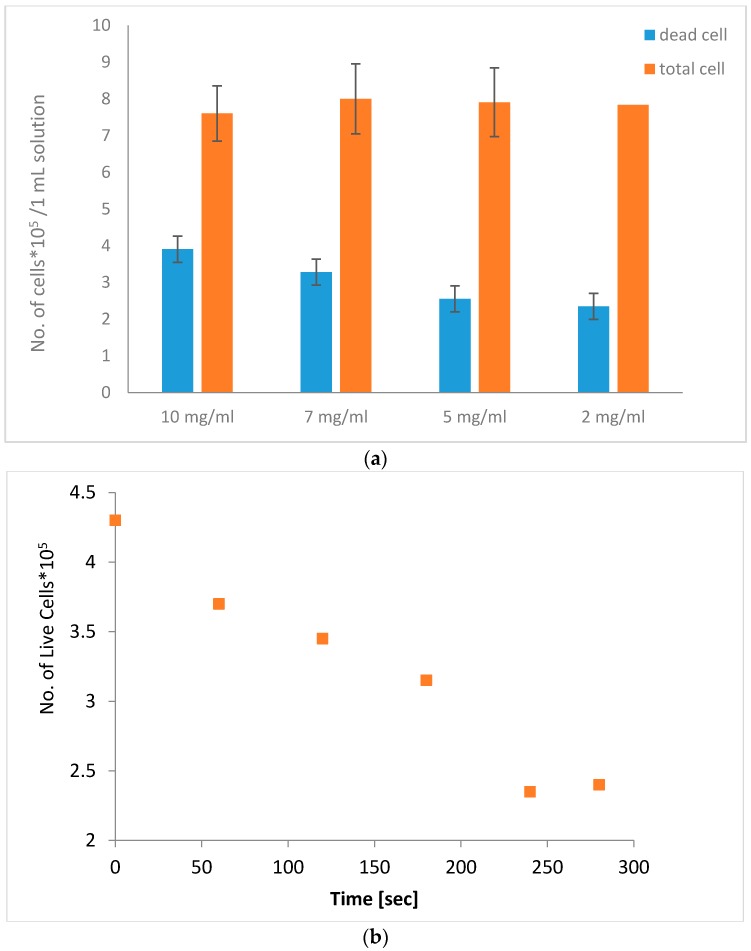
Number of cells in a 1 mL solution after AMF treatment for 5 min (**a**) with different particle concentrations (2 mg/mL–10 mg/mL); (**b**) The number of live cells in a sample with 10 mg/mL particles over a 5 min treatment.

We next examined the effect of different particle concentrations and AMF treatment on cell viability. Cell solutions with 2 mg/mL–10 mg/mL MNPs were treated with AMFs for 5 min, while measuring the solution temperature using T-type thermo-couples. During the experiment, the temperature was within the limits of 37 °C–38.4 °C. As shown in Figure 4a, we found that the 10 mg/mL solution reached 51% cell death, while 2 mg/mL induced only 30% cell death. The solutions with 7 and 5 mg/mL reached 41% and 32% cell death, respectively. This strengthens our assumption that the cause of cell death was not due to temperature changes but rather to a mechanical rupture of the cell membrane caused by particle motion. Next, we examined the effect of AMFs on the number of live cells in a cell sample with 10 mg/mL particles, while concurrently measuring near-surface temperature, throughout the 5 min treatment period. The number of live cells was measured every 60 s using a trypan blue assay. Figure 4b shows that the number of live cells over time reached ~50% of the initial amount by the end of the 5 min treatment period, indicating a gradual cell death mechanism.

### 3.2. Effect of Coated MNPs and AMF Treatment In Vivo

Nude mice aged 6 weeks received a subcutaneous injection of A431 cells and were monitored for tumor size. When the tumors reached a diameter of 4 mm–5 mm, the mice received an intravenous injection of either 50, 100, or 200 nm MNPs (*n* = 5 per group). Control mice (*n* = 4) were inoculated with the cancer cell line and received an injection of non-conjugated cetuximab only. All groups were exposed to seven treatments of 4 V, 4 Hz unipolar AMFs for 30 min, every other day (14 days in total). 

**Figure 5:**
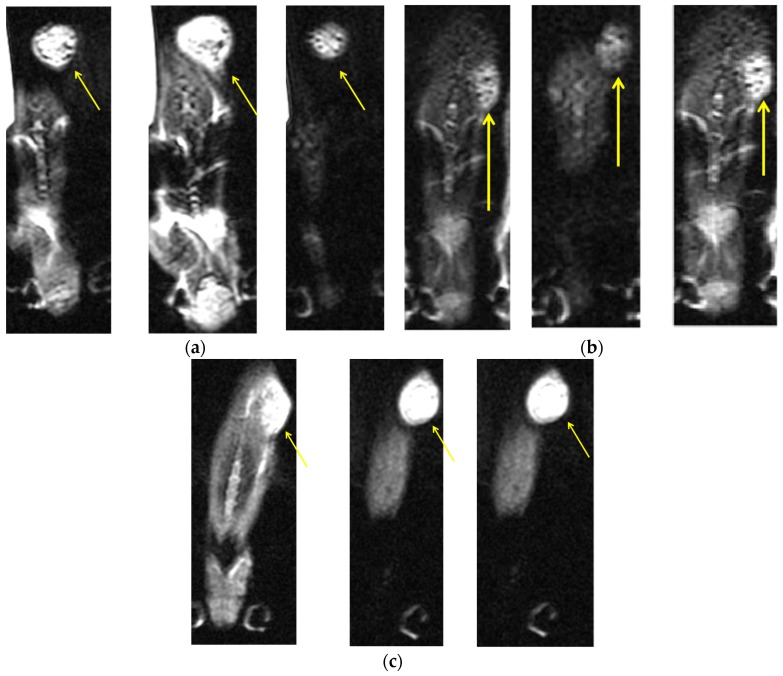
Axial contrast-enhanced axial T2-weighted MRI slices of a head and neck tumor in a representative mouse from each MNP size group, after three cycles of 30 min AMF treatments given on alternating days: (**a**) a mouse with 50 nm coated MNPs; (**b**) a mouse with 100 nm coated MNPs; and (**c**) a mouse with 200 nm coated MNPs. The yellow arrows indicate the tumor sites. Measurements were performed in 3 slices for each mouse (with 8.5 signal averages per position to improve signal-to-noise ratio) resulting in a total data acquisition time of 10 minutes. All MRI slices were 1.0 mm thick with a 0.15 mm in-plane resolution.

MRI images were obtained after three AMF treatments (Day 6). Superparamagnetic iron oxide MNPs, which are T2 contrast agents, reduce longitudinal (T1) and transverse (T2) magnetic relaxation time of non-bonded (water) protons, yielding a dark negative signal intensity in MRI images [27]. Figure 5a–c shows MRI images for the three MNP sizes in representative mice. The highest signal intensity was found for 200 nm particles (Figure 5c). Signal-to-noise ratios were 9, 5.54, and 11.032 for 50, 100, and 200 nm MNPs, respectively. For 50 nm particles, a large shift (imaged as signal voids) was observed within the tumor, demonstrating a negative T2 contrast [16,28] likely caused by the injected nanoparticles, suggesting their accumulation within the tumor site. The 200 nm MNPs show lower negative contrast, possibly due to the high accumulation of non-bonded protons in the tumor area, suggesting an enhanced effect of this MNP size.

**Figure 6:**
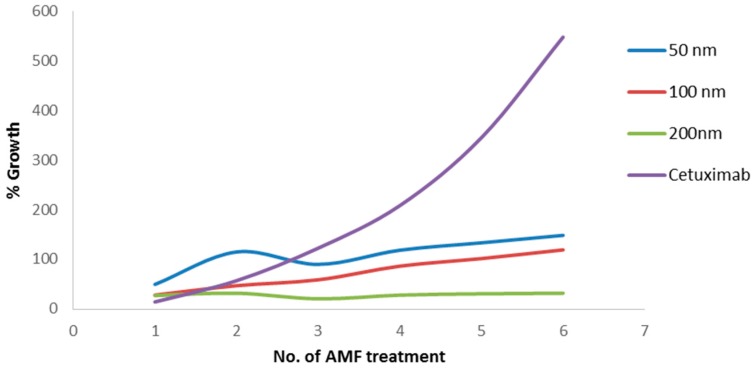
Percentage of tumor volume growth for each AMF treatment in mice injected with 50, 100, and 200 nm coated MNPs, as well as cetuximab alone. *N* = 5 for the MNP groups and *n* = 4 for controls at each time point.

To further examine the effect of the different MNP sizes and AMF treatment, tumor volume was measured in the treatment and control mice before each AMF treatment cycle. Figure 6 presents the average percentage of tumor growth throughout AMF treatments. We found that, after six AMF treatments, the tumor volume growth in the control group reached 548%, while the 50 nm group reached 148% growth, the 100 nm group reached 119% growth, and the 200 nm reached only 32% growth. A one-way ANOVA for tumor volume growth after the seventh measurement showed a statistically significant difference between groups (F(3,13) = 4.89, *p* = 0.017). An independent-sample *t*-test showed a significant difference between 200 nm MNPs and the cetuximab control (186.76% ± 164.15% vs. 398.28% ± 119.01% (mean ± SD), respectively; *p* = 0.023).

## 4. Discussion and Conclusions

In the present study, we show that treatment with cetuximab-coated MNPs leads to the death of human HNSCC in vitro. We demonstrated that this effect was due to MNP motion, and not heating, induced by AMF treatment. Moreover, in vivo, we found changes in MRI signal intensity in the tumor region for all three MNP sizes, with the highest intensity observed for 200 nm MNPs, as early as after three AMF treatments. The 200 nm MNPs also showed high efficacy in decreasing tumor volume after AMF treatments, as compared to the control. 

The key parameters affecting the MNP performance are surface chemistry, size (magnetic core and hydrodynamic diameter), and magnetic properties (magnetic moment, aggregation, and remanence). The surface chemistry is especially important for increasing the half-life in the blood stream by avoiding clearance by the RES [8,9]. Here, we used cetuximab coating to target the MNPs specifically to the tumor. The relaxation of MNPs is faster than nonbonding protons and appears as lower MRI signals [21]. Thus, the darker regions in the MRI images clearly demonstrate the presence of the MNPs at the tumor site, indicating the success of cetuximab targeting. 

Necrotic regions generally exhibit flowing of nonbonding protons, which results in a higher MRI signal [29,30]. Therefore, we postulate that the high signal intensities in the MRI images represent necrosis at the tumor site, likely caused by the AMF-induced MNP motion. Moreover, the MRI signal intensities for the different MNP sizes appear to be inversely related to tumor volumes for each size after treatment.

Due to the enhanced permeability and retention (EPR) effect of tumors, i.e., high-density and leaky vascular structure and ineffective lymphatic drainage, nanoparticles show increased accumulation at the tumor site [31]. Thus, we postulate that the EPR effect helped convey and spread the coated nanoparticles throughout the tumor, while the cetuximab coating subsequently bonded to the EGFR-expressing tumor cells. Furthermore, previous studies have shown that the MNP size is an important factor affecting retention at the tumor site [14,19], which supports the present findings. Specifically, the clearance rate from the tumor site is lower as the particle size increases in volume and mass [19], which may have caused the increased AMF-induced mechanical damage by 200 nm MNPs. In addition, the finding that mice treated with non-conjugated cetuximab show the highest tumor volume implies a lower retention time of free cetuximab at the tumor site. This further emphasizes the benefits of the combination of MNPs and conjugated cetuximab for effective, prolonged treatment. 

It is notable that, due to the higher surface area of 50 nm MNPs, the amount of cetuximab per injection volume is greater than for 200 nm MNPs. However, the smaller sized particles had a lower effect on tumor volume. This discrepancy, together with the fact that the therapeutic effect of cetuximab is typically slow [20], indicates that herein the cetuximab coating only served to target MNPs to the tumor, while the therapeutic effect itself was caused by MNP motion and force. 

We note that the current study was limited by the magnetic gradient, which decreases with distance, and by the restricted strength of the external field that can be applied in magnetic therapy. It has already been demonstrated that oscillating gradients can selectively destroy MNP-conjugated cells positioned in a saturated magnetic field [23]. Low-frequency dynamic magnetic fields have induced the rotation of MNPs 100 nm in diameter and, apparently, apoptotic cell death, due to the rupture of the lysosomal membrane [23]. Thus, as the external magnetic field applied here was relatively weak, a close proximity of mice to the coil was necessary. 

Altogether, our results indicate that cetuximab-coated MNPs activated by AMFs cause cell death purely by mechanical force. Moreover, 200 nm MNPs are the preferable size for treatment, showing a more potent therapeutic effect. This finding compliments our preliminary work that identified this MNP size as the most efficient for diagnostics with optimal speckle imaging capabilities [9]. It is notable that our AMF-induced MNP motion system is also more efficient compared to AMF-induced hyperthermia, due to its lower electric demand. In summary, we have demonstrated a new system for targeted cell death, using targeted magnetic nanoparticles in a mouse model for head and neck cancer. By incorporating advances in therapeutics, nanoscale particles, and fine imaging, MNPs have the potential to enable targeted and less damaging treatment for cancer, with greater effectiveness than ever before.
